# Lignin- and Cellulose-Derived Sustainable Nanofiltration Polyelectrolyte Membranes

**DOI:** 10.1021/acssuschemeng.4c08611

**Published:** 2025-01-29

**Authors:** Olawumi Sadare, Garyfalia A. Zoumpouli, Y. M. John Chew, Jannis Wenk, Bernardo Castro-Dominguez, Davide Mattia

**Affiliations:** Department of Chemical Engineering, University of Bath, Claverton Down, Bath BA2 7AY, United Kingdom

**Keywords:** biopolymer, cationic lignin, layer-by-layer, polyelectrolyte membrane, water treatment

## Abstract

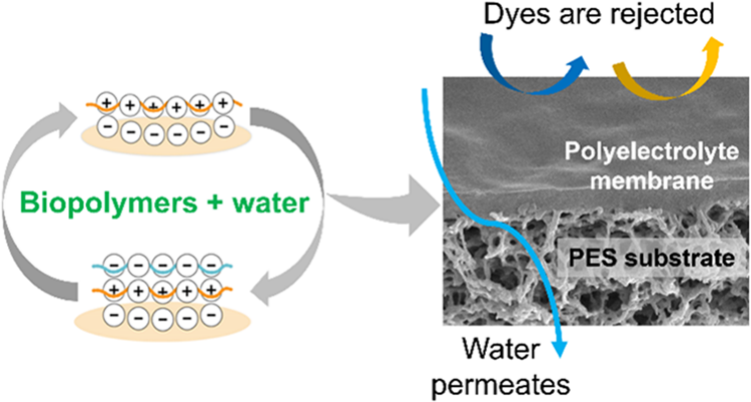

Nanofiltration (NF) polymeric membranes are typically made from fossil fuel-derived feedstocks and toxic solvents, requiring a shift to more sustainable materials. This study pioneers the use of two biopolymers–cationic lignin and sodium carboxymethyl cellulose–as polycation and polyanion, respectively, to fabricate a polyelectrolyte membrane (PEM) via the layer-by-layer method with water as the sole solvent and on a poly(ether sulfone) (PES) support. At a transmembrane pressure of 2 bar, the pure water permeance was 6 LMHB (L/m^2^ h bar) for 5 bilayers with a 96% rejection for positively charged methylene blue and 93% for negatively charged reactive orange-16, with a mass balance above 90%, indicating minimal adsorption on the membrane surface. The molecular weight cutoff (MWCO) of the PEM ranged from 300 and 620 Da, corresponding to a loose NF membrane. Additionally, the PEM demonstrated excellent stability after 30 days in deionized water, attributed to strong electrostatic interactions between the polyelectrolyte layers. This study demonstrates that effective NF membranes can be produced using sustainable biopolymeric materials and benign solvents. The efficient rejection of small, charged molecules makes the PEM membrane promising for protein removal, wastewater treatment, biotechnology, and pharmaceutical applications.

## Abstract 2

A stable, loose NF polyelectrolyte membrane has been fabricated using two biodegradable biopolymers and water as the only solvent, addressing the urgent need to replace fossil-based feedstocks and toxic solvents in membrane manufacturing.

## 1 Introduction

Nanofiltration (NF) membranes, with a molecular weight cutoff (MWCO) between 200 and 2000 Da, are rapidly growing in the membrane industry due to their high permeation flux and ability to reject small molecules at low pressures.^1−3^ These features make them ideal for removing heavy metals,^4,5^ dyes,^6,7^ multivalent ions,^8,9^ and other organic pollutants.^10−13^

Commercial NF membranes are currently manufactured using fossil fuel-derived feedstocks and toxic solvents.^14^ There is increasing urgency to switch to less toxic and more sustainable manufacturing alternatives,^15^ driven by regulatory frameworks, especially in the EU, which has banned several traditional solvents under REACH regulations.^16,17^ Proposed bans on fluorinated polymers could also impact the production of ubiquitous polyvinylidene fluoride (PVDF) membranes.^18^ This shift requires research into sustainable materials, moving beyond the current focus on enhancing fouling resistance, solute selectivity, chemical stability, and energy efficiency.^19^

A polyelectrolyte membrane (PEM) is a selective membrane formed by alternating layers of positively (polycation) and negatively (polyanion) charged polyelectrolytes, typically deposited using a layer-by-layer method onto a porous support for separation applications.^20,21^ The layer-by-layer (LBL) polyelectrolyte deposition method initially developed for ultrathin multilayer membranes, is now used for nanofiltration (NF) membranes.^22−24^ This method provides advantages in process control and simplicity compared to traditional methods like phase inversion, interfacial polymerization, and electrospinning, resulting in more consistent membrane quality.^25,26^ Research indicates that PEMs have the potential for high selectivity, permeance, improved ion conductivity, and versatility compared to other membrane types.^27,28^ However, further advancements are needed to translate these promising characteristics into commercially viable products. They have the potential to be cost-effective and customizable for applications such as fuel cells,^29^ water treatment,^30,31^ and biochemical and biomedical uses.^32^ PEMs can be fabricated using water as a solvent and modified to increase hydrophilicity, which helps resist fouling.^33^ Various polyelectrolyte pairs such as polydiallyl dimethylammonium chloride (PDADMAC) and poly(sodium 4-styrenesulfonate) (PSS),^34^ poly(allylamine hydrochloride) (PAH) and PSS,^35^ PAH and poly(acrylamide) (PAA),^21^ poly(ethyleneimine) (PEI) and PSS^36^ have been extensively studied for LBL techniques. Most materials are petroleum based, with limited research on sustainable polymers and nontoxic solvents.^37^

PEMs made from biopolymers could provide a more sustainable alternative to petroleum based.^38^ While lignin and cellulose are commonly recognized as cost-effective biobased materials,^39^ their potential as polyelectrolytes in PEM manufacturing remains largely untapped. Lignin, as the second most abundant natural compound in plants^40,41^ is stable in hydrolytic environments due to its aromatic structure but also contains hydrophilic groups,^42,43^ making it suitable for water purification membranes.^44,45^ Although naturally water-insoluble, lignin can be modified into water-soluble cationic lignin, enabling its use in PEM manufacturing, the purification of lignin remains an energy-intensive process, with major ongoing efforts aiming at overcoming this limitation.^46−48^

Recently, all-lignin polyelectrolytes–cationic lignin and lignosulfonate as polycation and polyanion, respectively–were used to fabricate a PEM.^49^ However, mixing cellulose and lignin would form more stable polyelectrolyte multilayers due to complementary interactions between their ionic group. Although sodium carboxymethyl cellulose (NaCMC), a polyanionic polysaccharide derived from cellulose, has been studied for its ability to reinforce nanocomposites in nanofibers,^39,50^ its use in PEM fabrication has not been previously reported. This suggests the potential for sodium carboxymethyl cellulose (NaCMC), a polyanionic polysaccharide derived from cellulose, to improve the brittle nature of lignin, leading to a more resilient membrane with enhanced mechanical properties. NaCMC is stable, water-soluble, and resistant to salt, acid, calcium, and high temperatures.^38^ Combining lignin and NaCMC polyelectrolytes via the LBL method has not yet been utilized in PEM manufacturing.

This study introduces a novel approach by incorporating cellulose-based layers with modified lignin, to enhance the mechanical strength and durability of membranes. For the first time, a polyelectrolyte pair of modified polycationic kraft lignin and polyanionic sodium carboxymethyl cellulose was used to fabricate a PEM via the LBL method, using water as solvent. These polyelectrolytes were alternately deposited onto a poly(ether sulfone) (PES) ultrafiltration (UF) support. The membrane’s structure and the attachment of functional groups were examined. The study assessed pure water permeance and the rejection of methylene blue (MB), methyl orange (MO), and reactive orange-16 (RO) dyes to evaluate the PEM’s suitability for water treatment in a crossflow setup. Additionally, the molecular weight cutoff (MWCO) was determined using different molecular weights of linear polyethylene glycol (PEG).

## 2 Materials and Methods

### 2.1 Materials

**Figure 1:**
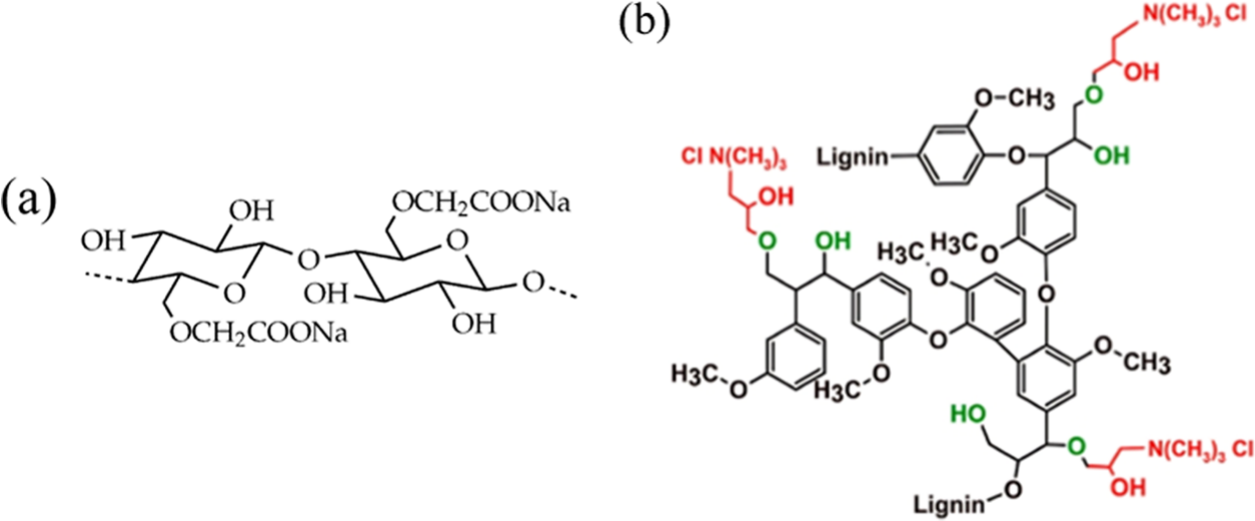
Chemical structures of (a) Polyanion: sodium carboxymethyl cellulose (NaCMC) and (b) Polycation: Cationic lignin.

Polyelectrolytes: Lignin alkali (*M*_W_ = ∼10,000 g·mol^–1^), sodium carboxymethyl cellulose (*M*_W_ = 250,000 g·mol^–1^), Synthesis: Ethanol (absolute, 99.8%), sodium chloride Bioxtra (purity, ≥99.5%), glycidyl trimethylammonium chloride solution (50% in water), sodium hydroxide (NaOH, pellets), sulfuric acid solution (H_2_SO_4,_ purity ≥97%), Testing: polyethylene glycols (PEGs) (*M*_W_ = 300, 400, 600, 1000, 2000 Da), and methylene blue (MB, *M*_W_ = 319.85 g·mol^–1^, positively charged), methyl orange (MO, *M*_w_ = 327.33 g·mol^–1^, negatively charged), and reactive orange-16 (RO, *M*_w_ = 617.84 g·mol^–1^, negatively charged) dyes were obtained from Sigma-Aldrich (Merck), United Kingdom. Poly(ether sulfone) (PES) ultrafiltration membranes (pore size 0.03 μm, diameter 90 mm, thickness 110–150 μm), were purchased from Sterlitech Corporation. Regenerated cellulose Ultracel membranes (MWCO, 1 KDa) were purchased from Sigma-Aldrich (Merck). Veolia Water Technologies produced deionized and ultrapure water (18.2 MΩ cm^–1^) using an ELGA Veolia LA758 water purification system. No additional purification steps were applied to the chemicals used. The chemical structures of sodium carboxymethyl cellulose and cationic lignin are depicted in Figure 1.

### 2.2 Preparation of Cationic Lignin

Cationic lignin was prepared as described by Peil et al.,^51^ Briefly, to prepare a 1 wt % solution of Kraft lignin, 1 g of lignin alkali was dissolved in 99 g of 0.2 M NaOH aqueous solution. Approximately 3.65 g of glycidyl trimethylammonium chloride (GTAC, 50% in water) was added dropwise into the solution containing lignin alkali and NaOH aqueous solution, and the reaction mixture was agitated at 70 °C for 1 h at a speed of 100 rpm, producing cationic lignin. Figure S1 represents the reaction of lignin alkali and GTAC to produce cationic lignin. The mixture was then neutralized by the addition of a 10 wt % solution of sulfuric acid. After that, dialysis was conducted over 3 days using 3 L of deionized water to remove all traces of low-molecular-weight lignin fractions and GTAC, and the water was changed twice daily. The dialysis was performed using SnakeSkin dialysis tubing with a MWCO of 3.5 kDa (22 mm × 35 feet dry diameter). Subsequently, the cationic lignin was recovered from the dialyzed product through vacuum filtration (filter paper: 0.45 μm pore size, Sterlitech), removing the insoluble lignin fraction. The recovered cationic lignin was allowed to dry at 50 °C in an oven for 24 h until a constant weight was achieved. Fourier Transform Infrared (FTIR) Spectroscopy, Zeta potential, and proton nuclear magnetic resonance (NMR) spectroscopy were used to analyze the synthesized cationic lignin.

### 2.3 Manufacturing of Polyelectrolyte Membrane (PEM)

**Figure 2:**
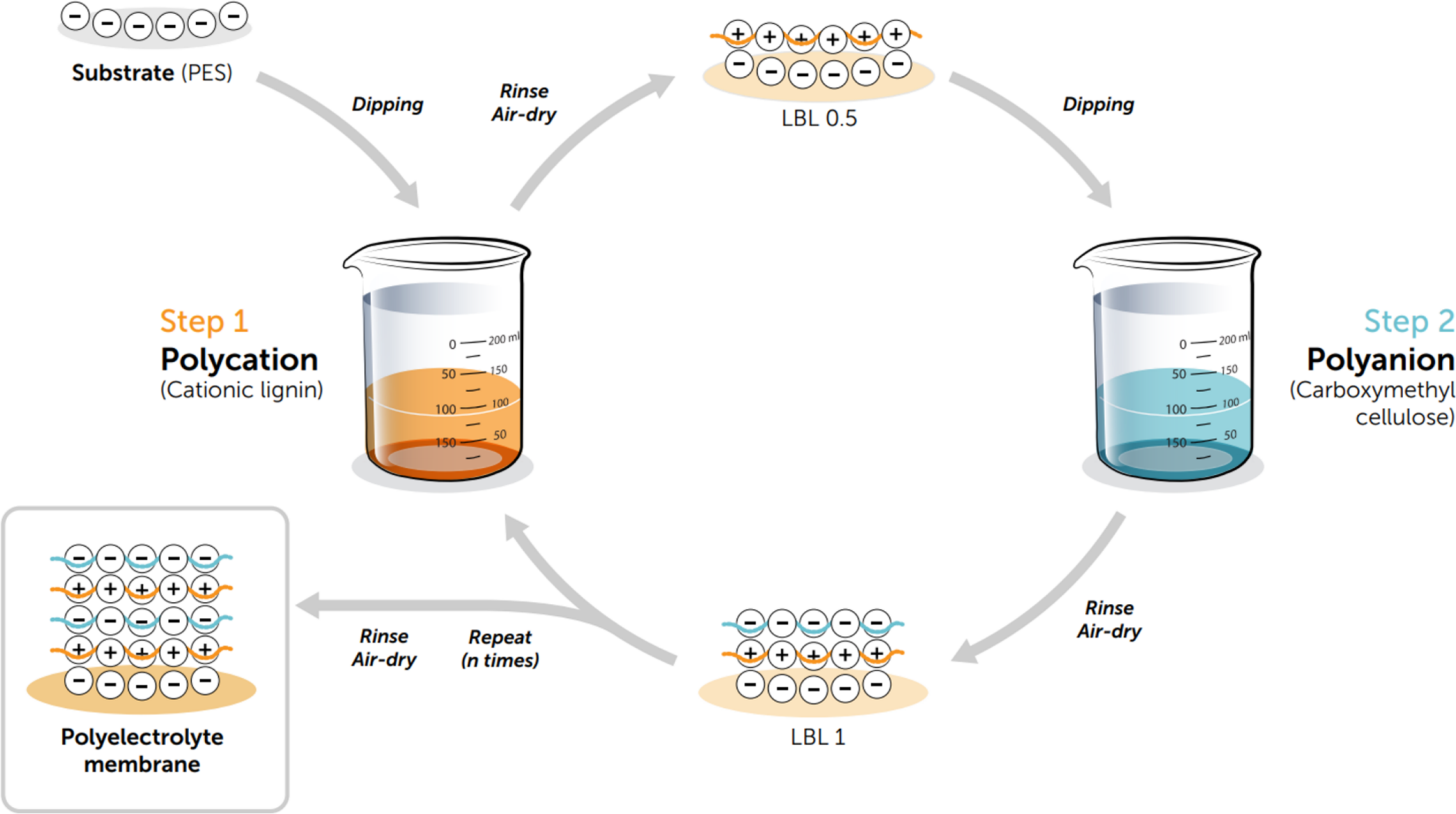
Schematic diagram of manufacturing of polyelectrolyte membrane.

Cationic lignin and sodium carboxymethyl cellulose (NaCMC) were used as polycation (positively charged polyelectrolyte) and polyanion (negatively charged polyelectrolyte), respectively, to manufacture the PEM membrane via LBL dip-coating. Initially, the PES substrates were cleansed by immersing them in a solution consisting of 10 wt % ethanol and water to remove any dirt. To improve the ionic strength and enhance the growth of the polyelectrolyte membrane, 50 mM of NaCl was used as the washing solution.^52,53^ Approximately 0.1 wt % of either cationic lignin or NaCMC was dissolved in 100 mL of the salt solution. A thermo Scientific Orion Star Orion VerasStar pH meter was used to determine the pH values of the cationic lignin and NaCMC solutions. The pH values of the solutions were adjusted to 6 and 4, respectively, using sodium hydroxide and sulfuric acid. This is to ensure that the polycation and the polyanions are completely ionized to form a polyelectrolyte membrane via electrostatic interactions. The polyelectrolyte membranes were fabricated dipping the PES UF support into the cationic lignin solution for 15 min and then rinsing it in the NaCl solution to remove weakly bound polyelectrolytes. To ensure consistent performance in the membranes’ manufacturing, the same amount of solution was used in the preparation of the membrane and a new solution was used for each membrane manufactured, while maintaining dipping time constant. Compressed air at 20 ± 2 °C was then blown gently to dry the membrane. The air-dried membrane was then dipped into the NaCMC solution for another 15 min, rinsed in NaCl solution, and allowed to air-dry as before. This dip-coating process formed one bilayer of polyelectrolyte, and the process was repeated until the required number of bilayers was completed. The schematic illustration of the manufacturing of polyelectrolyte membranes is depicted in Figure 2.

### 2.4 Characterization of Synthesized Cationic Lignin and the Polyelectrolyte Membranes

The attachment of chemical functional groups to the surface of the cationic lignin, and the attachment of chemical functional groups to the surface of the PEM membranes prepared from kraft lignin was examined using Attenuated Total Reflectance-Fourier Transform infrared (ATR-FTIR) (Frontier FTIR, PIKE Technologies Inc.), at wavelengths ranging from 600 to 4000 cm^–1^. The surface charge of lignin was measured using a Zetasizer (Malvern Panalytical, Nano Z). The kraft lignin and the cationic lignin were added to deionized water to form a suspension. The prepared kraft lignin and cationic lignin suspensions were loaded into separate sample cuvettes for analysis at 25 °C. A Nuclear Magnetic Resonance (NMR) spectrometer (Bruker Neo 500 MHz) was used to determine the structure of the prepared cationic lignin, by exploiting the magnetic properties of atomic nuclei at 25 °C. The cationic samples were dissolved in DMSO-d6, while kraft lignin was dissolved in sodium hydroxide. MestReNova software was used to export the data. The thermal stability of the manufactured polyelectrolyte membrane was assessed using the Setsys Evo 16/18.0 TG-DTA (Setaram). The experimental parameters were as follows: Temperature range of 45–850 °C, a heating rate of 20 K/min, and an argon flow rate of 50 mL/min for tests conducted under an inert atmosphere. Elemental composition analysis of the manufactured polyelectrolyte membrane was performed using an Hitachi SU3900 SEM with an Oxford Instrument Ultim Max EDX detector at a magnification of 10,000×, with an accelerating voltage of 10.00 kV and an SE input signal. Field Emission Scanning Electron Microscope (JEOL JSM-7900F FESEM) was employed to study the formation of the film layers on the PES membrane surface, as well as the morphology of the membranes’ cross-section. After being frozen in liquid nitrogen, polyelectrolyte membrane samples were fractured to prepare the membranes for cross-section analysis. The membranes were then dried in a vacuum oven at 30 °C overnight. After this, the samples were coated with 10 nm gold on a sputter coating machine to prevent charging. The membrane thickness was estimated by ImageJ 1.53t and an average of ten measurements on a SEM image was recorded for each membrane.

The surface charge of manufactured membranes was measured using an Electrokinetic Analyzer (Anton Paar Surpass3) having a cell for film measurement. The Helmholtz-Smoluchowski eq (eq 1) was employed to estimate the zeta potential of the polyelectrolyte membranes. The measurements were taken using a streaming potential approach, with a 100 μm capillary at pH 4–10, and mixed 0.01 M KCl salt solution.^54^
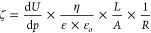
1The relationship between the streaming potential and the differential pressure ranging from 200–600 mbar (slope) is represented by . η represents the dynamic viscosity of the electrolyte and ε its dielectric coefficient. Permittivity is expressed as ε_o_, the streaming channel’s length is expressed as *L*, its cross-section is represented as *A*, and the measuring cell resistance is expressed as *R*.

### 2.5 Performance Evaluation of Polyelectrolyte Membranes

#### 2.5.1 Measurement of Pure Water Flux, Permeance and Dyes Rejection

The measurement of pure water flux (PWF), pure water permeance (PWP), and dye rejection were carried out using a crossflow setup to assess the performance of the manufactured polyelectrolyte membranes as previously described.^55^ The experiments were conducted at a feed flow rate of 3.1 L h^–1^, with a crossflow velocity of 0.590 m s^–1^. The temperature was maintained at 20 ± 2 °C, with transmembrane pressure (TMP) ranging from 2.0 to 6.0 bar. Deionized water was used as the feed to assess the membranes’ pure water permeance. The PWF, PFP, and dye rejections were calculated using eqs 2–4.
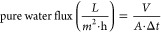
2
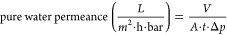
3where Δ*p* the operating transmembrane pressure (TMP) (bar), *V* the volume of permeate (L), *A* the membrane’s effective area (m^2^), and *t* the time (h) of the experiment. The diameter of the PES substrate is 47 mm, having a 0.00146 m^2^ effective filtration area. The rejection of MB, MO, and RO16 was performed in a crossflow setup with crossflow velocity of 0.590 m s^–1^ at a pressure of 2 bar for 1 h.
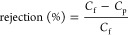
4where *C*_p_ is the permeate concentration (mg/L), and *C*_f_ the feed concentration (mg/L) which was equal to 3.5 mg L^–1^. A UV–vis spectrophotometer was utilized to analyze the absorbance of the dye solutions, at 663, 464, and 486 nm wavelength for MB, MO, and RO16 (Figure S1), respectively. The mass balance for these experiments was calculated using eqs 5 and 6.
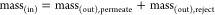
5
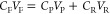
6where *C*_F_, *C*_P_, *and C*_R_ in g/L are the concentration of feed, permeate, and the retentate solutions, respectively. *V*_F_, *V*_F_ and *V*_F_ in L are the volume of the feed, permeate, and the retentate solutions, respectively.

#### 2.5.2 Measurement of Molecular Weight Cutoff (MWCO) of Polyelectrolyte Membranes

The manufactured polyelectrolyte membranes were used in the crossflow filtration setup. The MWCO was measured using a feed solution containing 1 g L^–1^ of multiple linear PEGs (polyethylene glycol) dissolved in ultrapure water. The experiment was conducted at a transmembrane pressure of 2 bar, for 1 h. The temperature was maintained at 20 ± 2 °C, and the flow rate of the feed solution was at 3.1 L h^–1^, with a crossflow velocity of 0.590 m s^–1^. The linear PEGs had molecular weights of 300, 400, 600, 1000, and 2000 Da. A commercial membrane (regenerated cellulose, Ultracel, MWCO 1000 Da) was used for comparison. The analysis of the PEG concentrations in the feed and the permeate was conducted using an Agilent gel permeation chromatography (GPC) instrument with 2 × PL aquagel–OH 30 8 μm, and a 300 × 7.5 mm (p/n PL1120–6830) columns in series. The analysis was performed with an injection volume of 20 μL, 1 mL min^–1^ flow rate, a working temperature of 35 °C, and ultrapure water as the eluent. Equation 4 was then used to determine the retention. The molecular weight cutoff of the PEM was analyzed by a precalibrated GPC after obtaining the standard calibration curve corresponding to the known concentration of the PEG standards. The molecular weight corresponding to 90% PEG molecule retention was determined by interpolating the data to calculate the MWCO.

## 3 Results and Discussion

### 3.1 Characterization of Kraft Lignin and Synthesized Cationic Lignin

The kraft lignin particles had a net negative surface charge in suspension (−33.05 mV). After modification to cationic lignin, the zeta potential changed to positive values (+22.93 ± 1.07 mV), in agreement with the literature.^51^ The increase in conductivity after modification, from 0.08 to 0.24 ± 0.05 mS cm^–1^, indicates a higher concentration of ions in the suspension, which is consistent with the introduction of cationic groups in the lignin structure.

**Figure 3:**
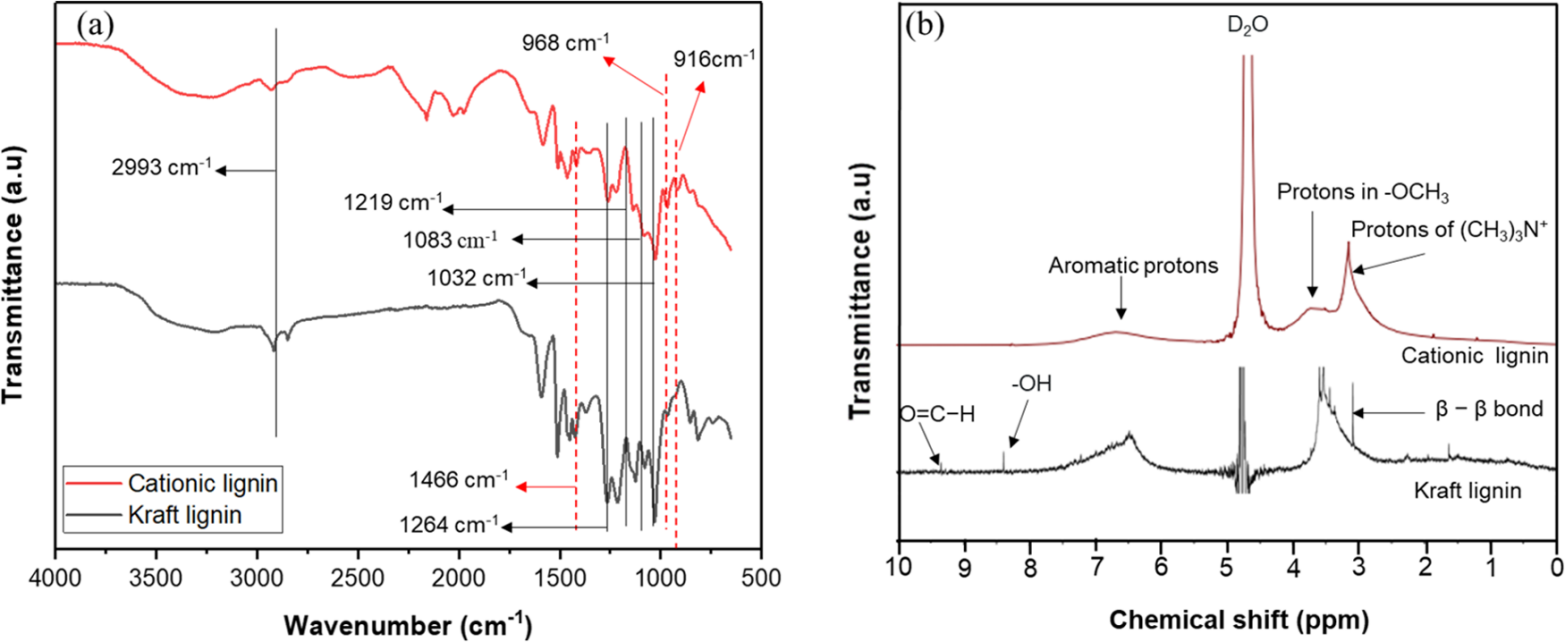
(a) FTIR (b) NMR spectra of synthesized cationic lignin and kraft lignin.

The FTIR of the cationic lignin also confirmed the success of the functionalization (Figure 3a), in agreement with the literature:^49^ The absorption band at 1083 cm^–1^ indicates an increase in hydroxyl groups, attributable to the OH groups in the lignin. The absorption peaks at 1264, 1219, and 1032 cm^–1^ indicate new ether linkages formed between GTAC and kraft lignin. Additionally, new peaks observed at 1466, 968, and 916 cm^–1^ correspond to the C–H vibrations of trimethylammonium, methylene, and methine groups of GTAC, respectively. The band intensity at 2933 cm^–1^ is associated with methyl and methylene groups.

The ^1^H NMR spectra further confirmed successful functionalization (Figure 3b), with the near elimination of peaks at 8.35 and 9.3 ppm in the cationic lignin spectra suggesting that phenolic groups have been substituted and aldehyde groups have undergone oxidation, respectively, consistent with what has been reported in the literature.^49,51^ The analysis also showed the continued presence of peaks corresponding to the primary lignin structure, indicating that this remains unchanged after synthesis. The aromatic protons are indicated by the broadband between 7.4 and 5.9 ppm, while the protons in the methoxy groups correspond to the broadband found between 4.05 and 3.00. The β–β bond coupling the lignin subunits peaks can be observed at 3.4 ppm.

### 3.2 Characterization of the Polyelectrolyte Membranes

**Figure 4:**
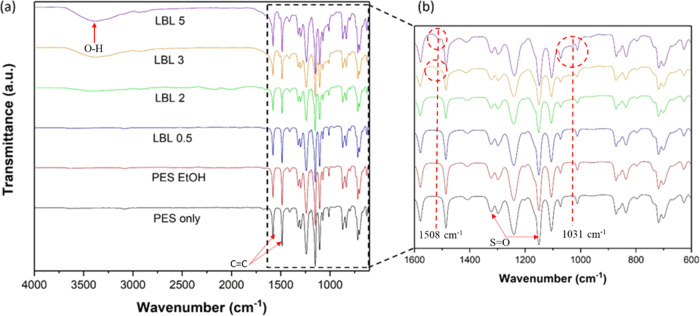
FTIR spectra of pristine PES ultrafiltration support and the polyelectrolyte membranes.

All PEMs as depicted in Figure 4 exhibit characteristic absorption peaks similar to those of pristine PES membranes. Notably, the peaks at 1580 and 1480 cm^–1^ are attributed to the C=C stretching vibrations of the benzene ring. The absorption peak at 1240 cm^–1^ corresponds to the ether linkage between phenyl groups, while the peaks at 1150 and 1100 cm^–1^ are indicative of the sulfone groups inherent in the PES porous structure. Also, the broad peak at 3400–3590 increased as the deposition of the LBL increased from 2 to 5 (LBL2-LBL5). This can be attributed to the −OH stretching of cellulose from the carboxymethyl cellulose^56^ and the cationic lignin.^57^ This was not observed in LBL 0.5, since there was no membrane formed yet. Figure 4(b) shows the formation of new absorption peaks at 1508 cm^–1^ for LBL3 and LBL5, which can be attributed to the existence of aromatic ring vibrations originating from the cationic lignin component. These peaks correspond to the aromatic C=C stretching vibrations in the lignin structure. Since this peak is not present in the spectrum of the poly(ether sulfone) porous support (LBL 0), they indicate the successful incorporation of cationic lignin onto the surface of the PES support membrane. Additionally, significant alterations in the absorption peaks at 1072 and 1012 cm^–1^ were noted for LBL2, LBL3, and LBL5. Between these two peaks, a new peak emerged at 1031 cm^–1^ for LBL3 and LBL5, indicating these peaks are associated with the C–O stretching vibrations in the carboxyl methyl cellulose component. These new peaks at 1508 and 1031 cm^–1^ signify the successful formation of PEM onto the surface of the PES UF support.

**Figure 5:**
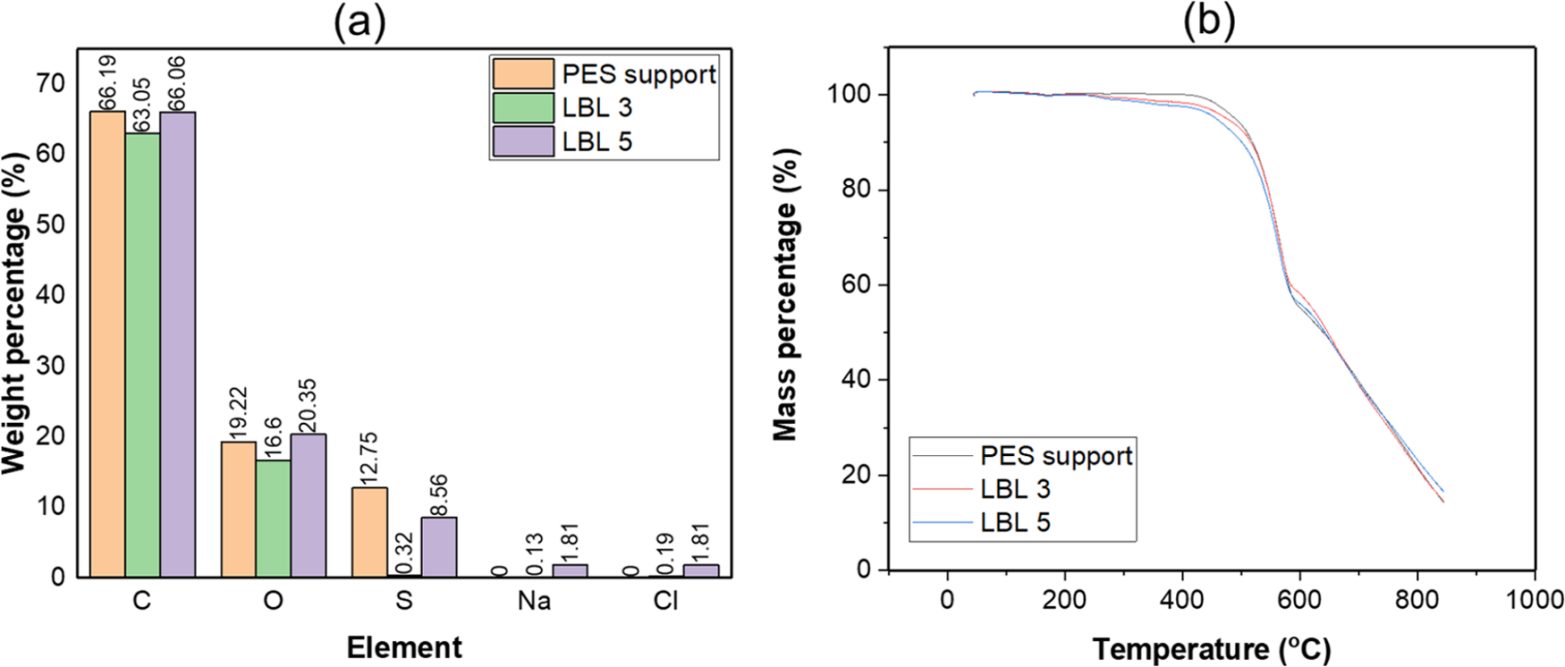
(a) EDX elemental analysis (b) TGA profile (c) dTG profile of PES support, LBL 3 and LBL 5 membranes.

Figures 5a and S2(a–c) illustrate the Energy-dispersive spectroscopy (EDS) elemental analysis of PES support and the fabricated polyelectrolyte membranes. The EDS analysis confirmed the presence of carbon (C), oxygen (O) and sulfur (S) in all the manufactured PEMs.^58^ The PES support displayed a high C (66.2%) and O (19.2%) content, consistent with its polymeric structure and sulfone (-SO_2_-) groups. The S content (12.8%) further confirmed the presence of sulfone functionalities.

In the LBL membranes, an increase in oxygen and a significant drop in sulfur content indicate effective coating of the PES support with polyelectrolyte layers. Sodium and chlorine, absent in the PES support, appear in the LBL membranes due to sodium carboxymethyl cellulose and cationic lignin. The sulfur content decreased further in LBL 3, with the oxygen content rising from 18.9% in LBL 3 to 20.6% in LBL 5, reflecting the increased bilayer thickness. Notably, the higher sodium (1.81%) and chlorine (1.81%) content in LBL 5 compared to LBL 3 highlights the accumulation of ionic species with additional bilayers. These findings further confirm the successful assembly and selective functionalization of the LBL membranes. The trace amounts of titanium and aluminum in the samples are likely to have originated from processing residues or contamination. Figure 5b presents the thermogravimetric analysis (TGA) of the PES support membranes (LBL 3 and LBL 5). All samples exhibit minor weight loss below 200 °C, attributed to the evaporation of water or volatile components. The PES support shows a distinct thermal degradation pattern characterized by a single sharp mass loss around 500–600 °C, indicating the decomposition of its polymer backbone, consistent with the behavior of poly(ether sulfone) materials.^59^ The LBL membranes exhibit slightly broader degradation peaks compared to the PES support, suggesting contributions from both the PES substrate and the added polyelectrolyte selective layers. Above 700 °C, a slight shift in the decomposition onset temperature is observed, particularly in LBL 5, indicating that the addition of more layers may enhance the thermal stability due to the interactions between the layers and the PES support.

**Figure 6:**
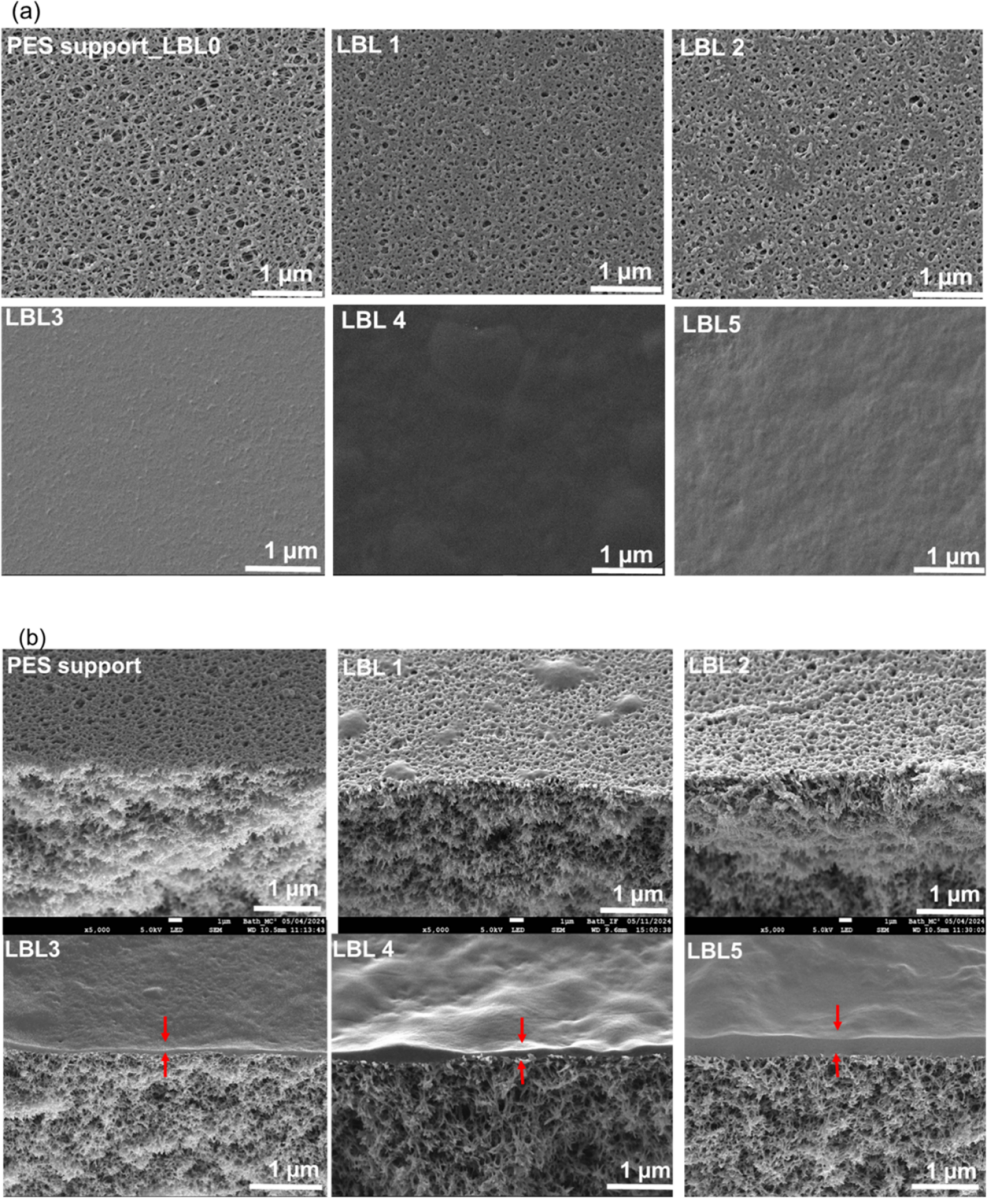
SEM micrographs of the top surface (top row) and the cross-section (bottom row) of the polyelectrolyte membranes.

SEM micrographs of the top surface and the cross-section of the PEMs show that, as the number of bilayers increases, the substrate’s pores are progressively covered, culminating in the formation of a continuous polyelectrolyte layer on the PES support in the LBL5 sample Figure 6. A similar trend is observed when looking at cross-sectional micrographs where, as shown by the red arrows in Figure 6 (bottom row), the thickness of the polyelectrolyte selective layer increases progressively with the number of bilayers, from 0.3 ± 0.1 μm for LBL3 to 1.1 ± 0.1 μm for LBL5. It was not possible to measure the thickness of LBL1 and LBL2 as no distinct layer was observed even at the maximum magnification. These findings align with the FTIR results (Figure 4 a), where the broad peak around 3400 cm^–1^ is more pronounced in LBL 5 than in LBL 3, indicative of an increased presence of hydroxyl groups from the polyelectrolytes.

### 3.3 Performance of the Polyelectrolyte Membranes

#### 3.3.1 Zeta Potential, Pure Water Permeance and Rejection of Dyes

**Figure 7:**
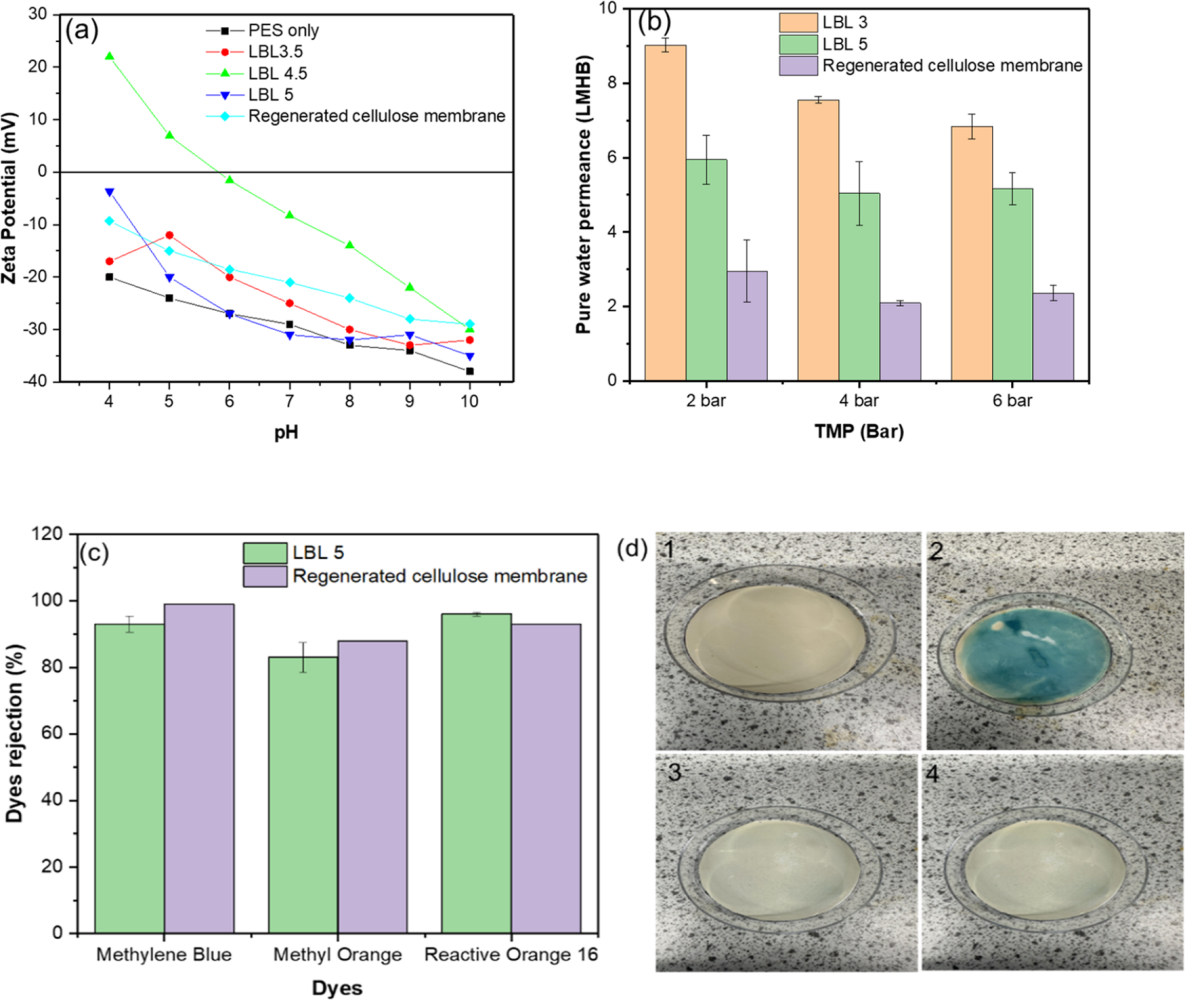
(a) Zeta potential and (b) pure water permeance at different TMPs, of LBL3, LBL5 and Regenerated cellulose membrane; (c) rejection of methylene blue, methyl orange, and reactive orange-16 by LBL5 PEM and Regenerated cellulose membrane; and (d) photographs of LBL5 PEM (1) before and after rejection of (2) MB (3) MO and (4) RO16.

The Zeta potential measurement of the PES UF support, the PEM, and the commercial membrane are reported in Figure 7(a), showing that the zeta potentials of the NaCMC-terminated PEM (LBL 5) and PES UF support were negative over the whole range of pH 4–10. The negative zeta potential for the LBL 5 could be a result of the ionization of the carboxylic group in the NaCMC backbone. It can also be observed that it was more negative than the cationic lignin(CL)-terminated membrane (LBL 3.5 and LBL 4.5). Following the adsorption of each polycation and polyanion, the zeta potentials are expected to switch between positive and negative. However, the zeta potential values in this study tended from positive toward negative in the case of LBL 4.5 as the pH increased from 4 to 10. The same trend was observed in Shan et al.,^60^ where the zeta potential remained consistently negative for the deposited multilayer The LBL 3.5 and 4.5 are expected to possess positive zeta potential values, being cationic-terminated PEMs. However, only LBL 4.5 had positive zeta potential values of +22 Mv at pH 4 and +6.7 at pH 5, then the zeta potential values were negative for the rest of the pH range. Meanwhile, the LBL 3.5 had negative zeta potential values all through the pH range, although higher than the zeta potential values for LBL 5. Furthermore, it could also be observed that the zeta potential of the membrane became less negative as the number of CL-terminated ends increased from 3.5 to 4.5. Even though there was an increase in the zeta potential values when the LBL was terminated with CL compared to when it was terminated with NaCMC, it was observed that the surface charge inversion did not occur as expected for the cationic polyelectrolyte-terminated membrane. This may be due to the interdiffusion of CL into the already deposited NaCMC, leading to the formation of membranes with negative charges.^52^ Scheepers et al.,^34^ also reported a significant change in the even–odd effect without sign reversal in the LBL-assembled membrane. Although, all membranes exhibited an odd–even effect, however, interdiffusion of NaCMC into the CL is less, resulting in more dominance of the negative charge of NaCMC in LBL5 PEM compared to other CL-terminated PEMs. The result also showed that the commercial membrane exhibited negative zeta potentials across the entire pH range as expected, primarily due to hydroxyl groups on the membranes’ surface.

The LBL3 PEM exhibits the highest pure water permeance (PWP) at TMPs of 2, 4, and 6 bar when compared to the LBL5 PEM and commercial membrane under the same pressures (Figure 7b). This increased permeance in LBL3 can be attributed to the thinner membrane formed on the surface of the porous support and hence reduced resistance to water flow compared to LBL5, as seen in the SEM cross-sectional micrograph in Figure 6. In the LBL assembly, each succeeding layer can add more structural complexity and, potentially, more water flow resistance.^61^ As the number of layers grows, the effective pore size decreases, while the tortuosity of the path that water molecules must pass through increases.^53^ Furthermore, each additional layer might contribute to a more densely packed membrane, decreasing the available pathways for water to flow through. The result in Figure 7b showed that the commercial membrane exhibited the lowest PWP at all TMPs investigated, from 2 to 3 LMHB, compared to the PEMs manufactured in this study. The lower permeance observed for the commercial membrane could be due to a more densely packed membrane, causing higher resistance to water flow.

It can also be observed that as the TMP increased, the pure water permeance of LBL3 decreased, due to the compaction of membrane layers, reducing the pore size and increasing resistance to water flow. A similar trend was observed for LBL5 and the commercial membrane. With LBL5 having more layers than LBL3, the membrane layers become more compact or tightly packed under increased TMP, further restricting water flow and reducing permeance.

The PEMs’ rejection performance was benchmarked against the same commercial regenerated cellulose UF membrane, using methylene blue, methyl orange, and reactive orange-16 dyes (Figure 7c). The molecular weights and charge of the three dyes tested are reported in Table 1.

**Table 1:** Mass Balance, Molecular Weights, and Charges of Different Dyes Rejected by LBL5 PEM

dyes	MB	MO	RO16
mass balance (%)	92.7 ± 1.7	99.9 ± 0.3	97.7 ± 0.3
M·wt. (g mol^–1^)	319.9	327.3	617.5
charge	positive	negative	negative

The LBL5 PEM had a high rejection of 96 ± 3% for the RO16 dye, with a MW of ∼620 Da and a negative charge. A high mass balance of 98.0 ± 0.3% indicates a very low level of adsorption onto the membrane surface, as confirmed by visual inspection post-testing (Figure 7d4).

MB and MO have similar molecular weights but opposite charges. As the LBL5′s top layer (carboxymethyl cellulose) is negatively charged (Figure 7a), one would expect a higher rejection for MO than for MB.^62^ However, the opposite was observed, with 93% rejection for MB and 83% for MO. In fact, the higher value for methylene blue can be attributed to some residual adsorption of the dye on the surface (Figure S3), as evidenced by the lower comparatively lower mass balance of ∼92.7 ± 1.7 compared to 99.9 ± 0.3 for MO and visual inspection post-testing (cfr. Figure 7d2). Nonetheless, even for MB the mass balance is above 90%, indicating a high level of rejection. The top surface of the membrane after MB rejection became rougher compared to the as-prepared PEM (Figure S3).

The commercial membrane showed rejection values of 99% for MB, 93% for RO16 and 88% for MO, with corresponding mass balance values of 88%, 99%, and 97%, respectively. A one mass balance value below 90% for MB indicates that a substantial part of the “rejection” is actually due to adsorption, as confirmed by the intense blue coloration of the membrane after testing (Figure 7d2). This is not the case for the other two, although the very high values reported here might decrease in the long-term steady-state behavior.

Considering all the above results, the MWCO of the LBL5 PEM is in the loose nanofiltration range of 300–620 Da, with comparable rejection performance to a commercial membrane but higher permeance.

#### 3.3.2 Molecular Weight Cutoff Measurement of PEMs

**Figure 8:**
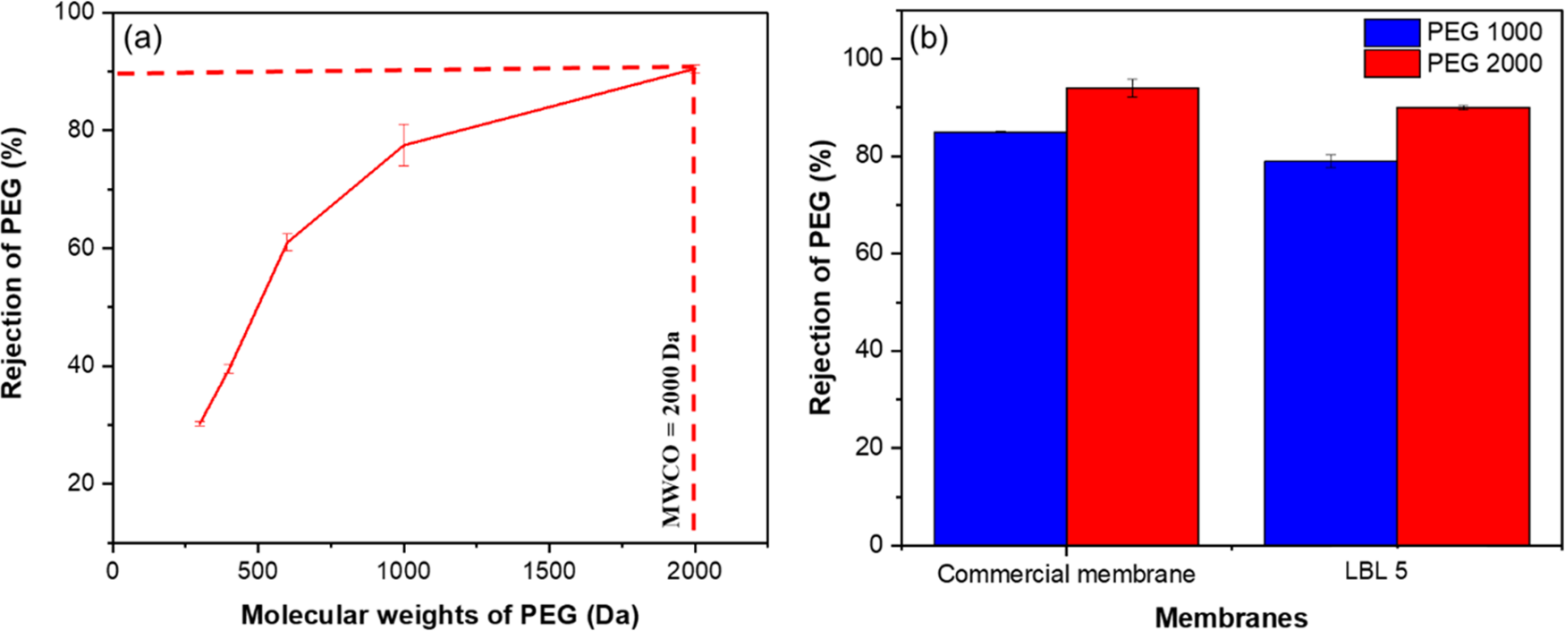
Rejection of PEGs of different molecular weights (a) 400, 600, 1000, and 2000 Da by LBL5 PEM; (b) Rejection of PEG 1000 and 2000 by a commercial membrane with nominal (MWCO = 1 KDa) and the LBL5.

The MWCO of the LBL 5 membrane was further assessed using neutrally charged, linear polyethylene glycol (PEG) of varying molecular weights (Figure 8a). The rejection percentages increased with the increasing molecular weight of PEGs, indicating a size exclusion mechanism for the rejection.

The low rejection of PEGs of molecular weight 400 and 600 Da might appear in contradiction with the high rejection of dyes of similar molecular weight reported earlier (cfr. Figure 7c and Table 1). However, the same apparent discrepancy has been observed previously in the literature and attributed to two main factors: First, the possible formation of dye aggregates due to the hydrophobic interactions between the aromatic rings of the adjacent dye molecules,^63^ and, second, the uncoiling of the PEG molecules which results in a high ease of penetration of membrane pores.^63^ In fact, comparative rejection tests using a dense polymer membrane and linear and branched PEGs of the same molar mass resulted in MWCO values of 1550 and 910 Da, respectively.^64^

The MWCO of the commercial Regenerated cellulose Ultracel membrane with a nominal MWCO of 1 KDa showed only 85 ± 0.1% rejection when using the same 1000 Da linear PEG used for the PEMs (Figure 8b). The same membrane exhibited a 94 ± 0.8% rejection for the 2000 Da PEG, compared to 90 ± 0.5% for the PEM.

The MWCO of the LBL5 PEM is higher than the 1159 Da value reported for an all-lignin polyelectrolyte membrane.^49^ However, that membrane required 10-layers to achieve sufficient rejection and stability. A MWCO of 2000 Da was also reported for a Poly(diallyldimethylammonium chloride) (pDAC) - lignin PEM membrane.^65^ Even though, the lignin used is a renewable material, the diallyldimethylammonium chloride is petroleum based. Furthermore, the results obtained in this study are comparable to those reported by Li et al.^66^ who fabricated a layer-by-layer (LBL) nanofiltration membrane using bamboo cellulose and chitosan as support and repeatedly sprayed sodium carboxymethyl cellulose onto the bamboo-cellulose film. Their membrane achieved a flux of 12.08 L/m^2^·h for salt rejection, which aligns closely with the flux of 11.7 L/m^2^·h obtained in this study. This similarity highlights the effectiveness of biobased materials, such as sodium carboxymethyl cellulose, in achieving competitive performance in nanofiltration membranes^65^ The MWCO of 2000 Da for the LBL5 membrane suggests linear PEGs overestimate the MWCO of the membrane. However, the rejection of smaller charged molecules such as dyes with the PEM’s MWCO in the range of 300–620 Da indicates a successful fabrication of a loose NF membrane.^65^

The performance of the LBL 5 PEM was further compared with polymeric NF membranes from the literature (Table 2). Although the PEM membrane well compares to the regenerated cellulose membrane, its pure water permeance tends to be lower than polyamide-based nanofiltration membranes, with values ranging as high as 130.4 LMHB for an amino acid–based PA/PES membrane,^67^ and as low as 7.7 LMHB for dendrimer trimesoyl amide amine (TMAAM) monomer on a polysulfone support.^68^ However, the LBL 5 PEM showed similar performance in terms of rejection for both dyes and PEG probes. Similarly, the LBL 5 PEM showed higher rejection but only slightly lower PWP compared to a polyamide membrane modified with diethanolamine (DEA) for removal of a neutral dye,^69^ which has a similar molecular weight of 314 g/mol to the methylene blue used in this study. These results conform to the well-known permeance-selectivity trade-off challenge of pressure-driven membrane process, with the added benefit, for the LBL 5 PEM of being manufactured sustainably while offering a competitive performance in terms of rejection and permeance.

**Table 2:** LBL 5 PEM Performance with Selected Sustainable and NF Membranes

membrane	dye or PEG	PWP (L/m^2^·h·bar)	dye rejection (%)	PEG MWCO (g/mol)	refs.
CL-lignosulfonate/HF	PEG 200–10,000	20		1159	(49)
pDac-lignin	PEG 400–20,000	1.6		2000	(65)
Amino-based PA/PES	Reactive orange-16	130.4	95	246	(67)
TMAAM-PA/PSF	PEG 1000	7.7		1000	(68)
	Methylene blue		98		
DEA-PA/PSF	Neutra red (314 g/mol)	18.0	81		(69)
regenerated cellulose membrane	PEG 1000			1000–2000	this work
LBL 5	Reactive orange-16	5.85	96		
	PEG 1000			1000–2000	

**Figure 9:**
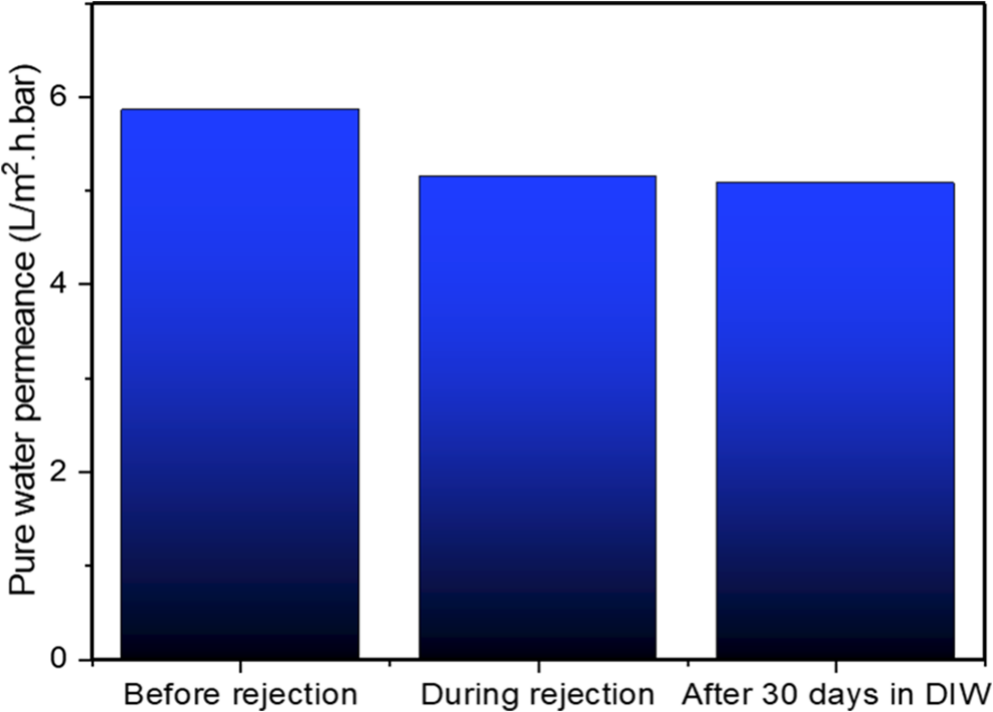
Stability of pure water permeance of LBL5 PEM after PEG test and after 30 days of soaking in water.

To evaluate the long-term performance of the LBL5 PEM in an aqueous environment, a stability test was conducted by measuring the PWP of a pristine membrane, after PEG MWCO and after soaking in deionized water for 30 days at 20 ± 2 °C (Figure 9). The pure water permeance decreased by ∼12% after the PEG test, due to fouling caused by the adsorption of PEG molecules onto the membrane surface. This is confirmed by an increased roughness of the PEM’s surface after the PEG rejection tests (Figure S4). After soaking for 30 days, the PWP was unchanged (difference less than 2%), indicating the membrane’s high stability in aqueous environments. SEM micrographs of the LBL5 PEM after 30 days in DI water at 20 ± 2 °C showed rough top surface morphology and cross-sectional structure (Figure S6). However, the PEM remains well-adhered to the porous support, with no visible defects. The change in surface morphology of the PEM compared to the as-prepared PEM in Figure S6 was a result of compaction during pure water and PEG rejection test. The observed stability can be attributed to the strong electrostatic interactions between the polyelectrolyte layers, which resist degradation in water. Stability in water is a desirable property for membranes used in water-based separation processes.

The PEMs’ performance remained stable under the mild acid condition, pH 5.5, of the PEG rejection tests (Figure S4). However, there is some evidence in the literature that exposure to solutions with a high concentration of strong acids or bases could significantly alter the membrane’s behavior during filtration.^70^ Furthermore, LbL assemblies formed via electrostatic interactions have exhibited some degree of reversibility under specific conditions, such as exposure to extreme pH, surfactants, or specific solvents that weaken or disrupt the interactions between the layers.^70,71^ Some studies have even the reversibility of layer-by-layer assembly, but not of a membrane, after a long-term use and removed the old layers using strong concentrated acid to replace these with new ones.^72^ Of course, the porous support must be able to tolerate this.

## 4 Conclusions

Cationic lignin and sodium carboxymethyl cellulose were utilized as a novel, more sustainable polyelectrolyte pair to fabricate a loose nanofiltration polyelectrolyte membrane on a PES porous support via layer-by-layer (LBL) assembly, using only water as solvent. The PEM was assessed for pure water permeance, dye rejection, and rejection of PEG with different molecular weights to evaluate its suitability for water treatment applications. The PEM’s performance was also favorably benchmarked against a commercial regenerated cellulose membrane (MWCO 1 kDa). The PEM consisting of 5 bilayers showed a similar high rejection to MB and RO16 dyes as the commercial membrane, while achieving a higher water permeance, with a MWCO in the 300 to 620 Da range, indicating a loose nanofiltration performance. Furthermore, SEM micrographs showed that the PEM exhibited excellent stability after being soaked in DI water for 30 days, with negligible change in pure water permeance. These findings demonstrate that an active separation layer can be fabricated with the LBL technique utilizing biobased polymers and only water as solvent. This study prioritized renewable and sustainable materials in membrane manufacturing, aligning with green chemistry principles by utilizing lignin (a sustainable biopolymer) and sodium carboxymethyl cellulose (a biodegradable material), employing water as the sole solvent to avoid hazardous organic solvents, and operating the filtration process under mild, energy-efficient conditions, all while achieving satisfactory membrane performance. This innovative approach holds promise for developing sustainable loose NF membranes with enhanced performance in applications such as the removal of natural organic matter, the purification of pharmaceuticals, and wastewater treatment.

## Supporting Information Available

The reaction of lignin and glycidyl trimethylammonium chloride to cationic lignin, EDX elemental analysis of membranes. SEM micrographs of the top layer and cross sections of the polyelectrolyte membranes before and after MB dye/PEG rejection, SEM micrographs and photos of the commercial Regenerated cellulose Ultracel membrane, SEM micrographs of the top layers and cross sections of the polyelectrolyte membranes after stability test (PDF)
