# Supplementary material for: Lignin- and Cellulose-Derived Sustainable Nanofiltration Polyelectrolyte Membranes
Source: ACS Sustain Chem Eng. 2025 Jan 29;13(5):2060–71. doi: 10.1021/acssuschemeng.4c08611 (PMC11816010; doi:10.1021/acssuschemeng.4c08611)
Supplement: Supplementary file 1 — sc4c08611_si_001.pdf [file sc4c08611_si_001.pdf]

Supporting Information

Lignin- and cellulose-derived sustainable nanofiltration polyelectrolyte membranes

Olawumi Sadare*, Garyfalia A. Zoumpouli, Y.M. John Chew, Jannis Wenk, Bernardo Castro-Dominguez, Davide Mattia*

Department of Chemical Engineering, University of Bath, Claverton Down, Bath, BA2 7AY, United Kingdom

Corresponding authors: os734@bath.ac.uk, dm294@bath.ac.uk.

Number of pages-6

Number of figures-6

Number of tables-0

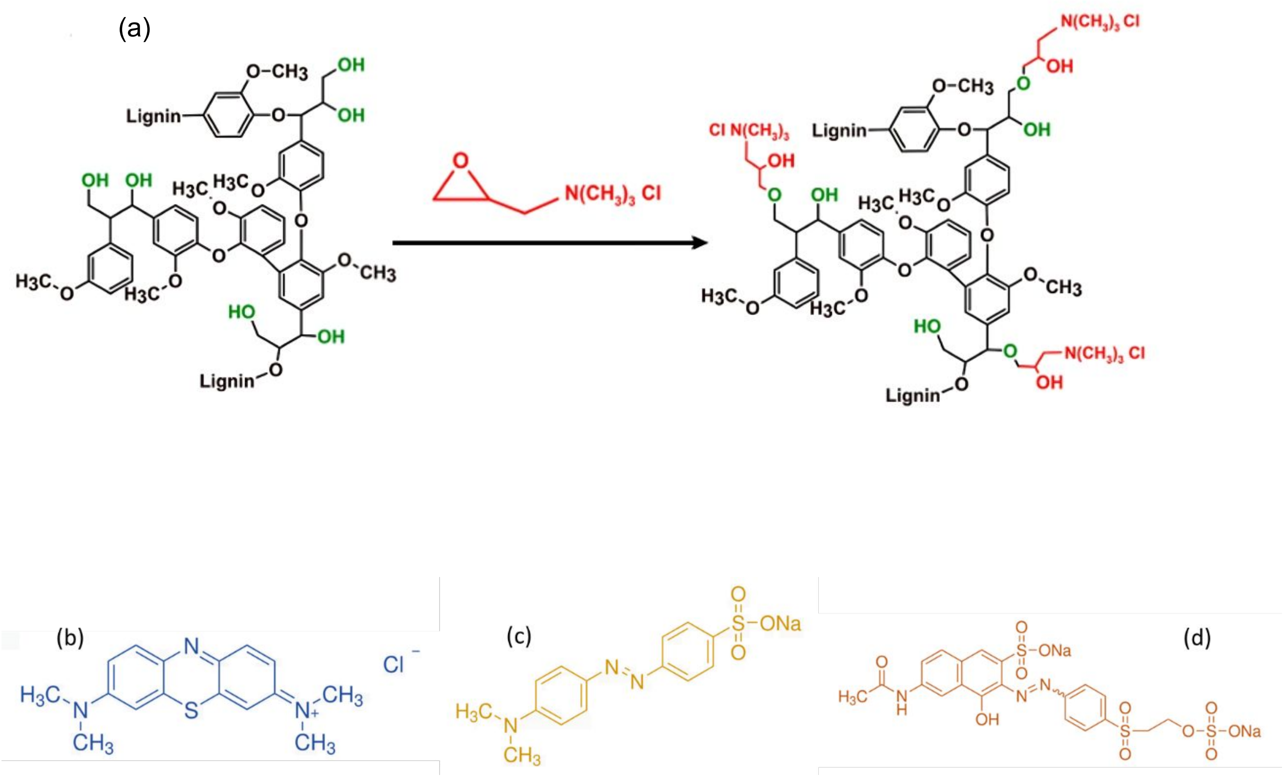

Fig. S1: (a) The reaction of lignin and glycidyl trimethyl ammonium chloride to cationic lignin (Watt et al., 2023). Chemical structures of the dyes (b) Methylene blue (MB) (c) Methyl orange (MO) (d) Reactive orange 16 (RO).

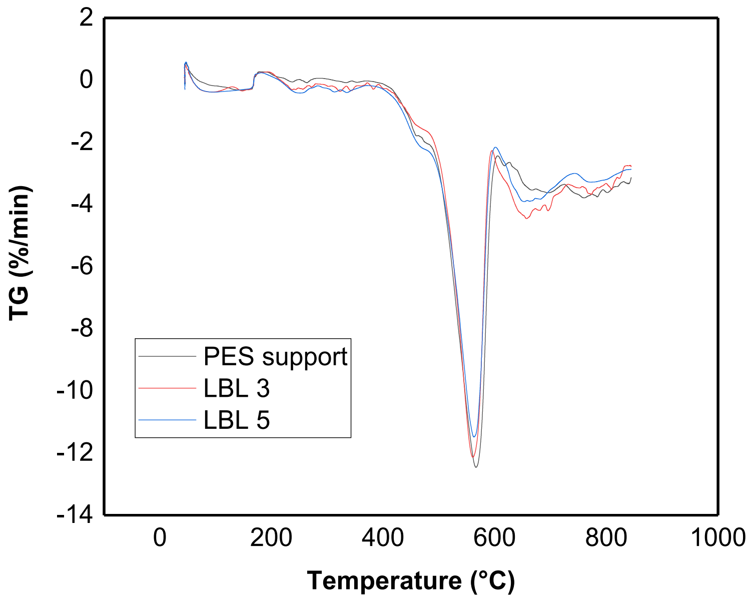

Fig S2: Derivative thermogravimetry (dTG) profile of PES support, LBL 3 and LBL 5 polyelectrolyte membranes.

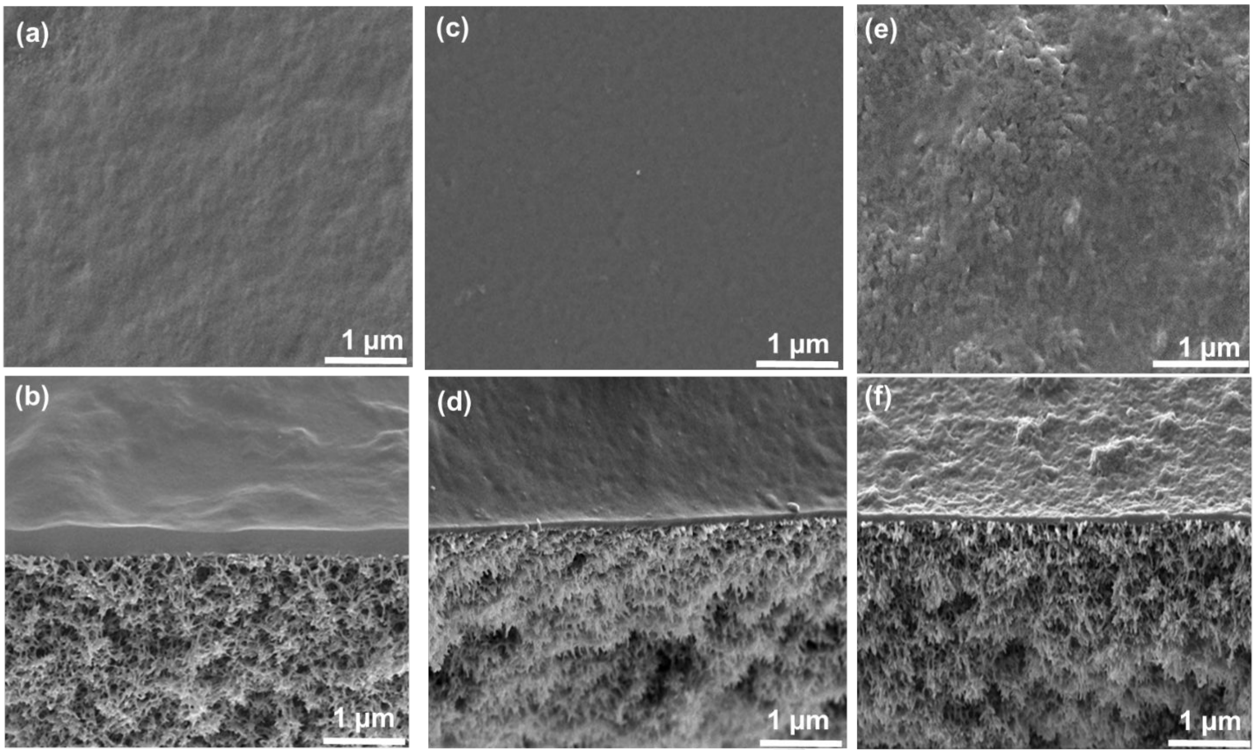

Fig. S3: SEM micrographs of the top layer and cross-sections of the polyelectrolyte membranes (a-b) before and (c-d) after MB dye rejection.

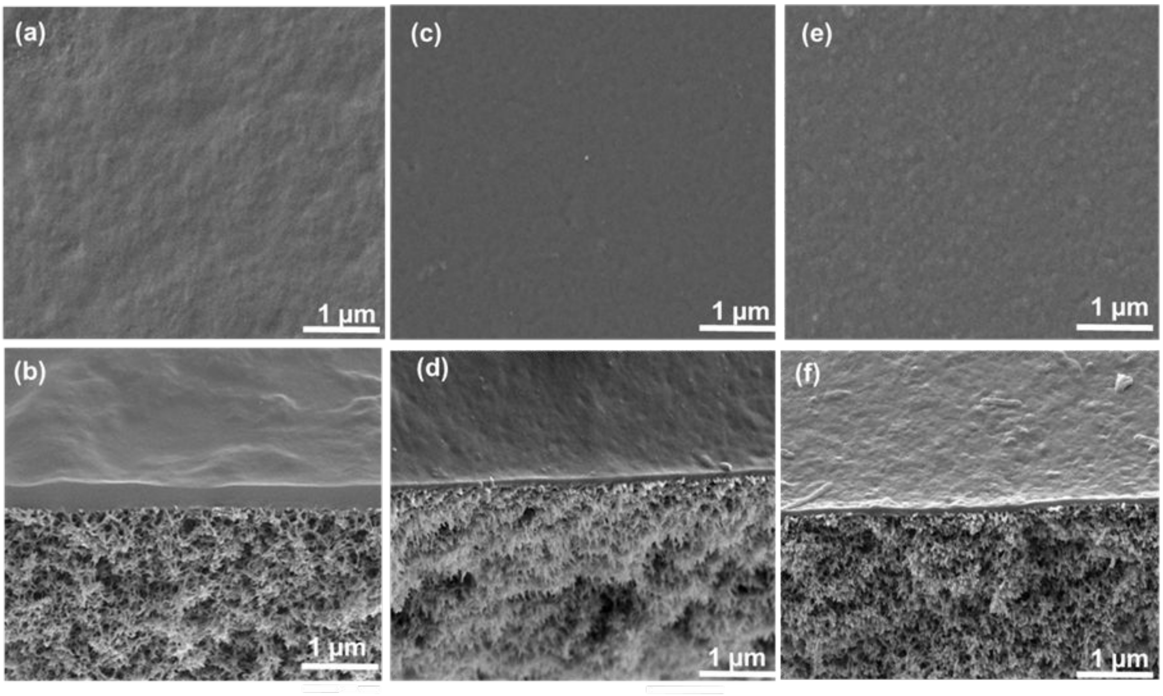

Fig. S4: SEM micrographs of the top layer and cross-sectional view of the polyelectrolyte membranes at 5k magnification (a-b) before (c-d) after pure water test (e-f) after PEG rejection

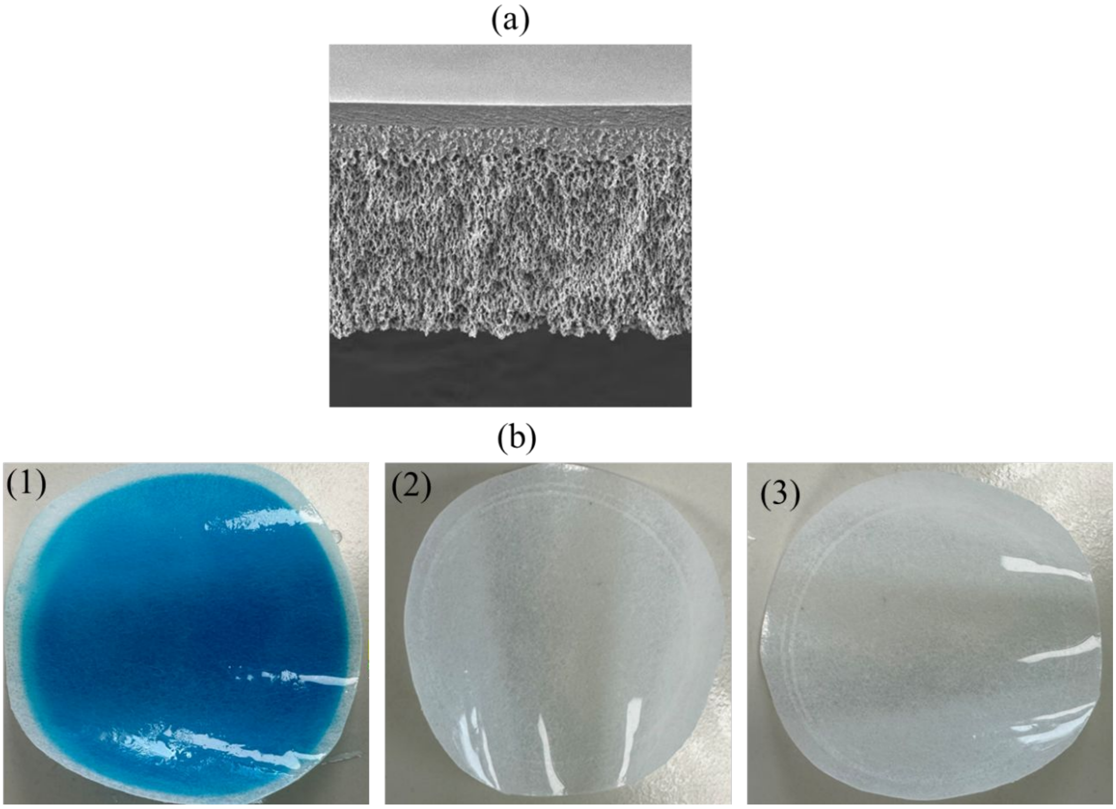

Fig. S5: (a) SEM micrographs of the cross-section of the commercial Regenerated Cellulose Ultracel membrane (b) Photographs of the commercial membrane after rejection of (1) MB, (2) MO and (3) RO16.

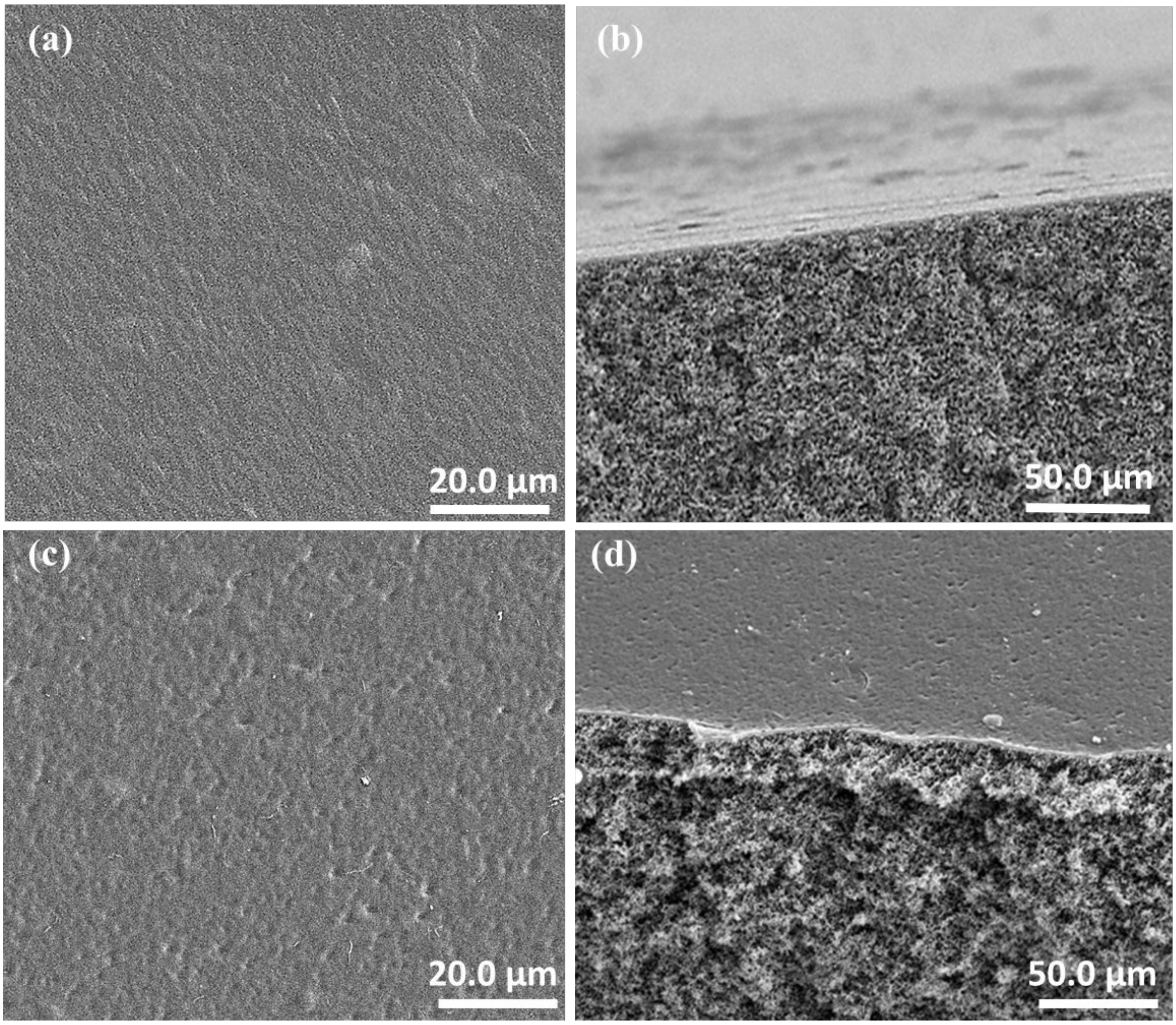

Fig. S6: SEM micrographs of the top layers and cross-sections of the polyelectrolyte membranes (a-b) As-prepared PEM and (c-d) after PEG rejection and stability test, respectively.
